# Discovery, optimization and biodistribution of an Affibody molecule for imaging of CD69

**DOI:** 10.1038/s41598-021-97694-6

**Published:** 2021-09-27

**Authors:** Jonas Persson, Emmi Puuvuori, Bo Zhang, Irina Velikyan, Ola Åberg, Malin Müller, Per-Åke Nygren, Stefan Ståhl, Olle Korsgren, Olof Eriksson, John Löfblom

**Affiliations:** 1 Department of Protein Science, Division of Protein Engineering, KTH Royal Institute of Technology, Roslagstullsbacken 21, 10691 Stockholm, Sweden; 2 Science for Life Laboratory, Department of Medicinal Chemistry, Uppsala University, Dag Hammarskjölds väg 14C, 3tr, 75183 Uppsala, Sweden; 3 Department of Immunology, Genetics and Pathology, Uppsala University, Uppsala, Sweden

**Keywords:** Protein design, Radionuclide imaging

## Abstract

Due to the wide scale of inflammatory processes in different types of disease, more sensitive and specific biomarkers are required to improve prevention and treatment. Cluster of differentiation 69 (CD69) is one of the earliest cell surface proteins expressed by activated leukocytes. Here we characterize and optimize potential new imaging probes, Affibody molecules targeting CD69 for imaging of activated immune cells. Analysis of candidates isolated in a previously performed selection from a Z variant *E. coli* library to the recombinant extracellular domain of human CD69, identified one cross-reactive Z variant with affinity to murine and human CD69. Affinity maturation was performed by randomization of the primary Z variant, followed by selections from the library. The resulting Z variants were evaluated for affinity towards human and murine CD69 and thermal stability. The in vivo biodistribution was assessed by SPECT/CT in rats following conjugation of the Z variants by a DOTA chelator and radiolabeling with Indium-111. A primary Z variant with a K_d_ of approximately 50 nM affinity to human and murine CD69 was identified. Affinity maturation generated 5 additional Z variants with improved or similar affinity. All clones exhibited suitable stability. Radiolabeling and in vivo biodistribution in rat demonstrated rapid renal clearance for all variants, while the background uptake and washout varied. The variant Z_CD69:4_ had the highest affinity for human and murine CD69 (34 nM) as well as the lowest in vivo background binding. In summary, we describe the discovery, optimization and evaluation of novel Affibody molecules with affinity for CD69. Affibody molecule Z_CD69:4_ is suitable for further development for imaging of activated immune cells.

## Introduction

The understanding of the immune system has long been an area of intense research. With the emergence of new immunotherapy approaches^1^, it is necessary to find novel non-invasive ways of studying the immune response in different diseases. Similarly, non-invasive tools to evaluate the involvement of the immune system in e.g. COVID-19 is urgently needed for understanding the progression of the disease and assessment of treatment effect. Furthermore, much is still unclear about the autoimmune response in many diseases, including type 1 diabetes (T1D), asthma, rheumatoid arthritis and psoriasis. The field of immunology is in dire need of clinically available biomarker for activated immune cells.

Cluster of differentiation 69 (CD69) is one of the earliest cell surface proteins expressed by activated lymphocytes^2–4^. It is also part of the lymphocyte proliferation and functions as a signal transmitting receptor^5^. Several other immune cell populations, including monocytes^6,7^ neutrophils^8,9^ and eosinophils^10^ express CD69 in all tissues explored so far. CD69 is constitutively expressed only by mature thymocytes and platelets, and it is not found on the resting circulating leukocytes in humans^2^. In fact, CD69 is also a marker for tissue resident T memory cells due to its ability to suppress tissue egress, by interacting with and internalizing S1P1^11^. Thus, CD69 is a general surface marker for activated immune cells in tissue, with limited presence in the circulation.

Currently, the only available high affinity binders for CD69 are antibodies. Antibodies can be radiolabeled for imaging, but to match the slow clearance, radionuclides with long radioactive half-life and associated radiation dose must be used. Radiation dose can be a limiting factor, especially when imaging vulnerable or young populations. Therefore, an ideal CD69 imaging agent must be significantly smaller than an antibody, while retaining high affinity and specificity. Hence, CD69 may be an interesting target for imaging probes due to the potentially low background binding in blood that otherwise could cause background signal.

Affibody molecules are small (6 kDa) scaffold proteins that consist of three alpha helices with 58 amino acids, which can be randomized to create large libraries from which potential binders may be isolated and further engineered^12^. The benefits of Affibody molecules also include their robustness and due to their small size, they clear out of plasma rapidly and penetrate into tissue. The biodistribution of radiolabeled Affibody molecules will directly influence their potential as in vivo imaging agents. Before embarking on further preclinical evaluation, we performed in vivo imaging in healthy rats to screen the Affibody molecules for lowest background binding in critical tissues (e.g. to optimize image contrast) as well as kidney retention (e.g. to choose the variant with optimal radiation dosimetry).

The aim of this study was to generate, characterize and optimize an Affibody molecule targeting CD69 for further development as an imaging agent of activated immune cells.

## Materials and methods

### Recombinant expression of the first generation and affinity matured CD69-binding Z variants

The gene encoding the first generation Z_CD69:2_ or the affinity matured variants Z_CD69:#_ was subcloned into three different *E. coli* expression vectors based on pET22b (GenScript Biotech Corp) under control of a T7 promoter. All constructs had an N-terminal hexahistidine tag and contained the gene encoding Z_CD69:#_. One construct had an additional C-terminal cysteine, and in another, Z_CD69:2_ was followed by a (G_4_S)_3_ linker and then an albumin binding domain (ABD) derived from streptococcal Protein G. Thus, the three expression products from these constructs were H_6_-Z_CD69:#_, H_6_-Z_CD69:#_-Cys and H_6_-Z_CD69:#_-(G_4_S)_3_-ABD. The ligated vector was transformed into *E. coli* BL21(DE3) cells (Merck) for expression using standard protocols. The recombinant proteins were purified using HisPur Cobalt Resin (#89,966, Thermo Scientific) according to the manufacturer’s instructions.

### Affinity maturation of the first generation CD69-binding Z variant

An affinity maturation library was designed with the distribution of varied amino acid positions shown in Supplementary Table 1.

DNA encoding the library was obtained from Twist Bioscience. Transformation of the DNA into *E. coli* BL21(DE3) cells (Merck) gave 6 × 10^8^ transformants. 96 clones were picked at random and sequenced. The sequencing showed that the library was highly functional, and no sequence occurred more than once, except for the original variant Z_CD69:1_, which occurred 6 times.

Induced recombinant *E. coli* cells were washed with 1xPBSP buffer (10 mM PBS, pH 7.4, containing 0.1% Pluronic F127). Cells were resuspended in PBSP containing biotinylated hCD69. The mix was incubated on a rotamixer at RT for 1 h, followed by extended washes with ice-cold PBSP, and resuspended in 150 nM human serum albumin (HSA)-Alexa 647 conjugate and 0.5 μg/ml streptavidin conjugated with R-phycoerythrin (SAPE) (Invitrogen) or neutravidin conjugated with Oregon Green 488 (NAOG) (Life Technologies), followed by incubation on ice for 30 min. The cells were subsequently washed with ice-cold PBSP and resuspended in ice-cold PBSP for sorting in a MoFlo Astrios EQ flow cytometer (Beckman Coulter) or analysis in a Gallios flow cytometer (Beckman Coulter). The *E. coli* library cells were sorted in a MoFlo Astrios EQ cell sorter (Beckman Coulter).

The sort gate was set to sort out the top fraction of cells displaying Z variants (typically 0.1%) showing the highest R-phycoerythrin or Oregon Green 488 to Alexa Fluor® 647 fluorescence intensity ratio. Bacteria were sorted into a 1.5 ml tube containing LB medium and chloramphenicol. The sorted cells were incubated for 1 h on rotamixer at 37 °C and thereafter inoculated to 50 ml LB medium with chloramphenicol for overnight cultivation.

### Affinity analysis by surface plasmon resonance

Human serum albumin (HSA; #A3782, Sigma), hCD69 (R&D Systems) and murine CD69 (mCD69; #8469-CD-025, R&D Systems), were each diluted in 10 mM Sodium Acetate, pH 4.5 and immobilized on CM5 chip surfaces using 1-ethyl-3-(3-dimethylaminopropyl) carbodiimide hydrochloride (EDC)/ N-hydroxysuccinimide (NHS) coupling chemistry for use as immobilized targets in a Biacore T200 instrument (GE Healthcare). The surfaces were inactivated using ethanolamine prior to binding studies. One surface was activated/inactivated for blank subtraction.

A first screening was performed by injecting 100 nM of the respective Z variant over the respective immobilized targets for 120 s, followed by running buffer for 300 s before regeneration of the surfaces. Z_CD69:2_ in fusion with ABD was first injected for non-covalent and directed capture on immobilized HSA, followed by injection of respective target molecule. Either 10 mM HCl or 10 mM glycine–HCl pH 2.5 were used for regeneration in the experiments. The five Z variants selected in the affinity maturation procedure expressed as described above were injected in the concentration range 1 nM, 5 nM, 25 nM, 50 nM and 100 nM, in triplicates over all four surfaces.

### Circular dichroism spectroscopy

Thermal stability and refolding after heat-induced denaturation was measured for each H_6_-Z_CD69:#_ variant using circular dichroism spectroscopy. All measurements were performed on a Chirascan™ instrument (Applied Photophysics Ltd, Surrey, UK). The thermal stability was determined by following the ellipticity at 221 nm during variable temperature measurements (5 °C/min from 20 °C to 100 °C). After the heat-induced denaturation, the samples were cooled to RT and left for 15 min before measuring ellipticity at 20 °C from 195 to 260 nm in five technical replicates.

### DOTA conjugation of the CD69-binding Z variants

All the six tested Z variants were expressed in the format H_6_-Z_CD69:#-_Cys and buffer exchanged to PBS after purification. For DOTA conjugation the Z variants were treated with an equimolar amount of tris-(2-carboxyethyl)phosphine (TCEP) to 1 mg/ml (118 mM) and incubated for 20 min at 37 °C. Following the incubation with TCEP, a tenfold molar excess of 1,4,7,10-tetraazacyclododecane-1,4,7,10-tetraacetic acid (DOTA) maleimide was added and the sample was incubated at 37 °C for 3 h. The progression of the conjugation was monitored by matrix-assisted laser desorption/ionization (MALDI).

The DOTA-conjugated H_6_-Z_CD69:#_ was diluted using acetonitrile (ACN) with 0.1% trifluoroacetic acid (TFA) to a final concentration of 20% ACN, and then sterile filtered through a 0.2 µm filter prior to injecting into HPLC for purification on a Semi-Prep C18 column. A gradient from 20 to 56% ACN at 2.5 ml/min over 30 min was used. 500 µl fractions were collected for the peaks, and subsequently analyzed by MALDI. The purified DOTA conjugated Z variants were freeze-dried prior to radiolabeling.

MALDI was used to verify DOTA conjugation during the conjugation as well as after HPLC purification. All samples were analyzed on a 4800 MALDI (Applied Biosystems). One µl samples were mixed with 1 µl α-cyano-4-hydroxy-cinnamic acid matrix (Bruker Daltonics), added to the MALDI plate and substituted with another µl of matrix before loading the plate in the MS.

### Radiolabeling of the CD69-binding Z variants

The DOTA-conjugated Affibody molecules were further radiolabeled with Indium-111 to enable evaluation of in vivo biodistribution of the final constructs.

All reagents were purchased from Sigma‐Aldrich (analytical grade or higher) and used without further purification, unless stated otherwise. Indium-111 chloride (370 MBq/ml) was purchased from Curium. Ammonium acetate buffer (0.2 M, pH 5.5) was prepared in plastic flask (200 ml, HDPE low metal resin from Nalgene) by dissolving 0.771 g ammonium acetate in 200 ml water. pH was adjusted by adding a few drops of glacial acetic acid (> 99%, TraceSELECT), while measuring with a pH meter (Mettler Toledo). Chelex 100 sodium was added to the buffer, which was allowed to stand in a refrigerator (4 °C) overnight. A NAP-5 column (GE Healthcare illustra) was pre-treated with 3 ml 1% bovine serum albumin (BSA) and rinsed with excess 6 ml phosphate buffered saline (PBS). Protein LoBinding Eppendorf tubes (1.5 ml) were used for the reaction and elution collection.

Analytical HPLC consisted of a VWR Hitachi Chromaster 5110 pump, a Knauer UV detector 40D, a Bioscan Flow count equipped with an Eckert & Ziegler extended range module 106, a Bioscan B-FC-3300 radioactivity probe, a VWR Hitachi Chromaster A/D Interface box and a Vydac 214MS, 5 µm C4, 50 × 4.6 mm column. The eluents were: A = 0.1% TFA in water, B = 0.1% TFA in acetonitrile, with an elution gradient from 5 to 70% B over 15 min at a flow rate of 1.0 ml/min.

For radiolabeling, a hydrochloric acid solution (0.02 M) of ^111^InCl_3_ (Curium) was buffered with sodium acetate or HEPES, and pH was adjusted to 5.0–5.5. Thereafter, the respective DOTA-conjugated Z variant (3–14 nmol) dissolved in phosphate buffer was added. Additionally, a previously described non-binding “scramble” Affibody molecule Z_TAQ_, raised against bacterial *Taq* DNA-polymerase and conjugated with DOTA, was similarly radiolabeled with Indium-111 for use as negative control ^13^.

The reaction mixture (in total 400–500 µl) was incubated at 80 °C for 30–60 min. The crude product was purified on solid phase extraction cartridge (HLB, OASIS; eluted with 1 ml of 50% ethanol) or NAP-5 column (elution with 200 µl PBS × 5). See Supplementary Table 2 for details of the conditions for each variant. The difference in details is due to progressive optimization of the procedure for this class of Z variants. All radiochemical yields (RCYs) were isolated yields and purity was measured by HPLC.

The original variant, ^111^In-DOTA-Z_CD69:2_, was evaluated for in vitro binding to activated immune cells to verify retained binding capacity after radiolabeling (Supplementary Data).

### Assessment of biodistribution by SPECT-CT imaging

All procedures involving animals were in compliance with the ARRIVE guidelines, approved by the Animal Ethics Committee of the Swedish Animal Welfare Agency and carried out in accordance with the relevant national and institutional guidelines (“Uppsala university guidelines on animal experimentation”, UFV 2007/724).

^111^In-DOTA-Z_CD69:#_ variant biodistribution in healthy rats was assessed by Single-photon emission computed tomography (SPECT-CT) imaging. Sprague–Dawley rats (n = 15 in total, n = 3 per Z variant, male, weight 318 ± 43 g) were injected in the lateral tail-vein with approximately 8 MBq of indium-111 labeled Z variant (^111^In-DOTA-Z_CD69:2_: 3.9 ± 1.6 MBq; ^111^In-DOTA-Z_CD69:4_: 12.6 ± 4.6 MBq; ^111^In-DOTA-Z_CD69:6_: 5.5 ± 1.3 MBq; ^111^In-DOTA-Z_CD69:8_: 8.0 ± 2.6 MBq; ^111^In-DOTA-Z_CD69:12_: 9.9 ± 2.6 MBq, ^111^In-DOTA-Z_TAQ_: 2.7 ± 1.4 MBq).

The examination by SPECT-CT (nanoSPECT, Mediso, Hungary) was carried out immediately post injection (0 h) as well as 3 h, 20 h, 48 h and 72 h post injection. For each scan, the animal was anesthetized and positioned by a whole-body CT acquisition. Next, a 20-min whole body static SPECT examination was performed. Some the animals were then moved to a preclinical PET/MRI scanner (nanoPET/ 3 T MRI, Mediso, Hungary) via the detachable bed, and examined by MRI. During each imaging session, the rat was sedated by gas anesthesia (sevoflurane 5% initially and afterwards 3% to maintain anesthesia) through a facemask. Temperature was maintained by warm air supply integrated in the scanner bed.

CT acquisition was performed before all SPECT examinations for anatomical co-registration (semicircular multi-field-of-view; duration per bed 7:46 min; 3 rotations; scan length 231.66 mm; 480 projections; binning 1:4; 50 kV; 600 µA; voxel size 0.25 × 0.25 × 0.25 mm). SPECT were acquired using the energy windows suitable for indium-111 emission (energy map: primary peak 245.35 keV, secondary peak 171.30 keV), and reconstructed using an iterative algorithm (iterations/subsets 48/3).

SPECT image analysis for all time-points was performed using the Nucline software (Mediso, Hungary). The kidneys, liver, heart, lung and muscle were segmented directly on SPECT images using co-registered CT projections as support. The uptake values were decay corrected to the time of administration and converted to standardized uptake values (SUV) by correcting for animal weight and administered amount of ^111^In-DOTA-Z_CD69:#_ variant.

Additionally, variant ^111^In-DOTA-Z_CD69:2_ was evaluated in an in vivo model of allograft rejection (Supplementary Data).

### Ethical approval

All applicable international, national, and/or institutional guidelines for the care and use of animals were followed.

## Results

### Characterization of a first-generation CD69-binding Z variant

Analysis of candidates isolated in a previously performed selection^14^ from a Z variant *E. coli* library to the recombinant extracellular domain of human CD69, identified one cross-reactive Z variant (Z_CD69:1_) with affinity to murine and human CD69. However, when expressed as soluble protein in *E. coli,* the yield for Z_CD69:1_ was lower than what has been generally observed for other Affibody molecules. Using homology alignment against previously reported Z domains, four potential amino acid replacements in the scaffold regions of helix three were identified and mutated. These substitution mutations in Z_CD69:1_ was S42A, E43N, S46A and S54A, and are all outside of the posited binding surface of the three-helix domain polypeptide. The resulting mutated Z variant is denoted Z_CD69:2_ herein. The expression yield for Z_CD69:2_ was at least 50-fold higher than that of Z_CD69:1_. Note that no mutations have been made to the CD69 binding motif of Z_CD69:1_, so Z_CD69:1_ and Z_CD69:2_ share the same binding motif sequence.

**Figure 1:**
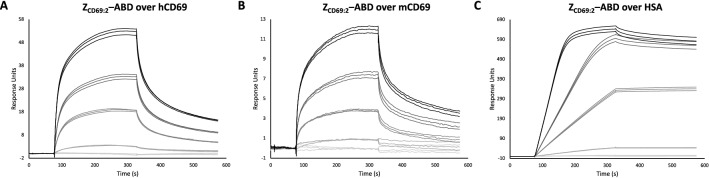
SPR sensorgrams showing the binding of H_6_-Z_CD69:2_-ABD to (**A**) human CD69, (**B**) murine CD69 and (**C**) human serum albumin at different concentrations (1 nM, 5 nM, 25 nM, 50 nM and 100 nM).

The Z_CD69:2_ was subcloned and produced in *E. coli* as part of three different constructs: H_6_-Z_CD69:2_, H_6_-Z_CD69:2_-Cys and H_6_-Z_CD69:2_-ABD. Purification using IMAC resulted in pure proteins of correct size, as assessed by SDS-PAGE. The interactions to CD69 and albumin were analyzed in SPR-based biosensor assays and H_6_-Z_CD69:2_-ABD was shown to bind HSA, hCD69 and mCD69 (Fig. 1). H_6_-Z_CD69:2_-ABD had an affinity of in the range of 50 nM to human and murine CD69. For PET tracers, the affinity (K_d_) is generally inversely correlated to the ability to detect lower number of receptors (B_max_). Thus, improvement of affinity is crucial for detecting small changes in CD69-presenting immune cells. In order to achieve a higher affinity, affinity maturation was carried out on Z_CD69:1_ i.e. the original version of Z variant Z_CD69:2_.

The secondary structure content, thermal stability and refolding was analyzed using circular dichroism spectroscopy. It was demonstrated that Z_CD69:2_ had the expected $\mathrm{\alpha }$ helical structure, had melting temperature typical for Affibody molecules and refolded after temperature denaturation.

### Affinity maturation of the first generation CD69-binding Z variant

**Figure 2:**
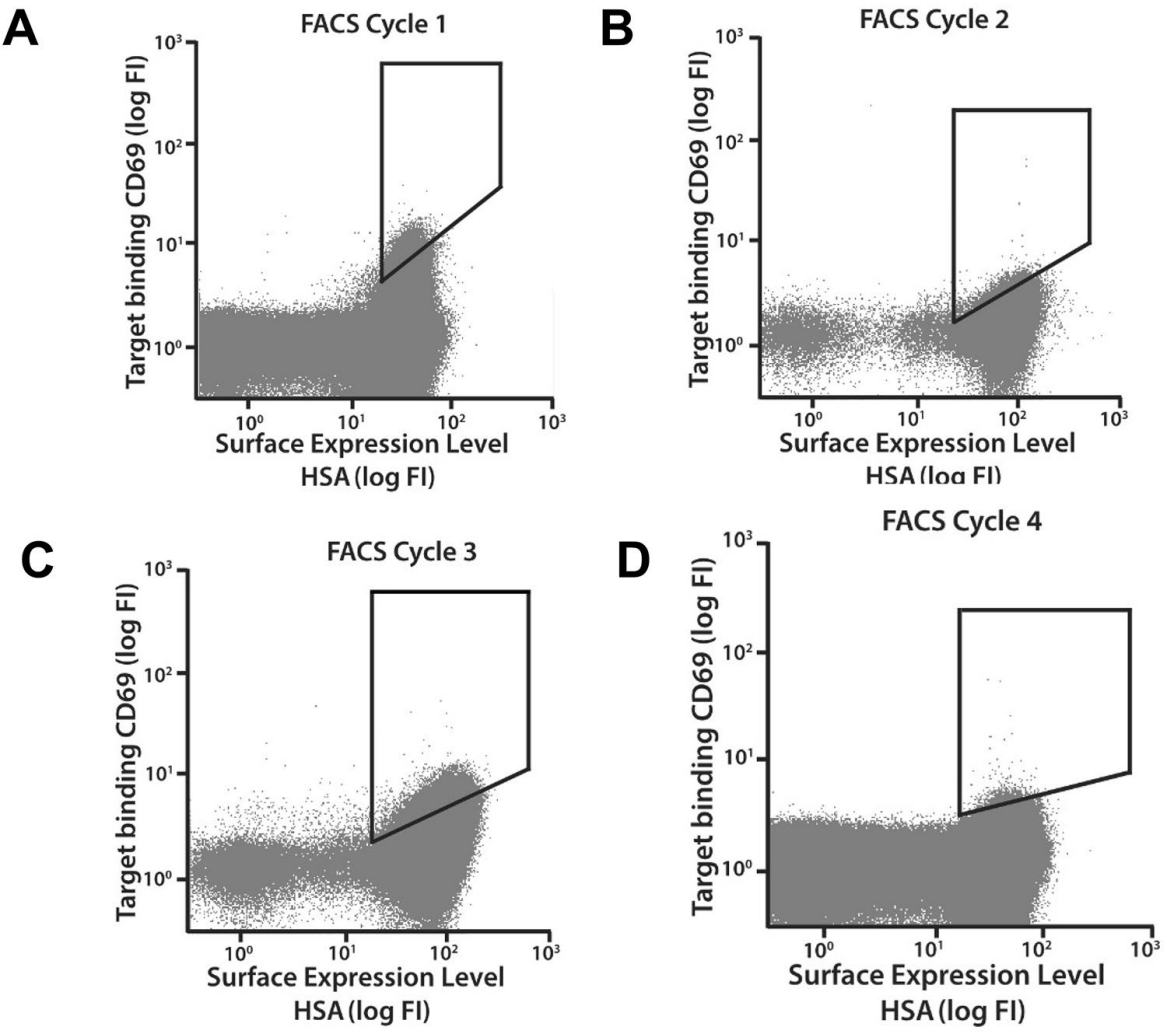
Dot plots of the E. coli-displayed affinity maturation library during each of four consecutive cycles of FACS (**A**–**D**). X-axes: fluorescence intensity corresponding to surface expression level as measured 25 by incubation with fluorescently labeled HSA. Y-axes: fluorescence intensity corresponding to labeled CD69 binding. Gates used for FACS are indicated.

For isolation of Z variants with an improved affinity for human CD69, an affinity maturation library was designed, subcloned to the *E. coli* display vector, expressed on the surface of *E. coli* and finally subjected to three rounds of fluorescence-activated cell sorting (FACS) with alternating rounds of amplification by cell growth. Briefly, cells were incubated with biotinylated hCD69 followed by extensive washes and then incubated with fluorescently labeled streptavidin for subsequent fluorescence-mediated detection of cell-bound hCD69 as well as fluorescently labeled HSA for monitoring of surface expression levels. The incubation of secondary reagents and HSA was performed on ice in order to reduce the dissociation rate of bound hCD69. After an additional washing, the labeled cell library was screened and sorted in a flow cytometer. Selection stringency in terms of target concentration, sorting parameters and sorting gates was increased with each sorting round and typically, the top 0.1% of the library demonstrating the highest ratio of target binding to surface expression, was gated and isolated for amplification and subsequent rounds of sorting. One advantage with cell-based selection systems is the straightforward monitoring of the obtained enrichment throughout the selection process. The visualization of the target-binding properties of the library in the flow cytometer revealed an enrichment of hCD69-positive clones in each sorting round (Fig. 2). After up to three rounds of FACS, isolated cells were spread on semi-solid medium and 44 randomly picked variants were sequenced. Clones appearing more than once among the sequences were selected for further characterization.

### Subcloning and expression of affinity-matured candidates

Z variants that were selected for further characterization were subjected to site-directed mutagenesis in analogy to the creation of Z_CD69:2_ from Z_CD69:1_, i.e. introduction of the scaffold position mutations S42A, E43N, S46A and S54A, creating mutated Z variants named Z_CD69:4_, Z_CD69:6_, Z_CD69:8_, Z_CD69:10_ and Z_CD69:12_. Genes encoding these five Z variants were each subcloned into tree different *E. coli* expression vectors. All constructs had an N-terminal hexahistidine tag and the corresponding gene encoding the Z variant sequence in question. For each Z variant, one of the three constructs had a C-terminal cysteine, and one of the three constructs encoded the H_6_-tagged Z variant in fusion with a linker and ABD. Thus, the three expression products for each Z variants were H_6_-Z_CD69:#,_ H_6_-Z_CD69:#-_Cys and H_6_-Z_CD69:#-(_G_4_S)_3_-ABD, wherein Z_CD69:#_ corresponds to one of the five Z variants listed above. The sequences for the H_6_-Z_CD69:#-_Cys variants are shown in Supplementary Fig. 1.

### Biochemical characterization of affinity matured CD69-binding Z variants

**Figure 3:**
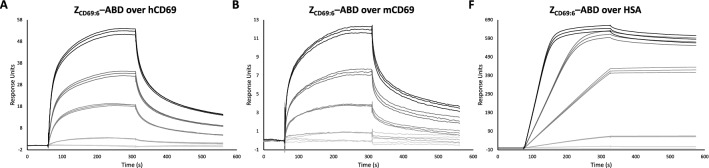
Representative series of SPR sensorgrams showing the binding of affinity maturated Affibody molecule, in this case H_6_-Z_CD69:6_-ABD, to (**A**) human CD69, (**B**) murine CD69 and (**C**) human serum albumin at different concentrations (1 nM, 5 nM, 25 nM, 50 nM and 100 nM).

The interactions to CD69 and albumin were analyzed in SPR-based biosensor assays and the five tested Z variants were found to have an increased or similar affinity towards hCD69 compared to the first-generation variant, Z_CD69:2_. A representative sensorgram from the SPR analysis is given for Z variant Z_CD69:6_ in Fig. 3, and the calculated K_d_ values for interaction with human and murine CD69 are given in Table 1.

**Table 1:** Affinity constants for tested Z variants from SPR.

Tested Z variant in the format H_6_-Z_CD69:#_-(G4S)3-ABD	K_d_ (nM)		
	vs. hCD69	vs. mCD69	vs. HSA
Z_CD69:2_	52	67	0.27
Z_CD69:4_	34	34	0.17
Z_CD69:6_	51	46	0.09
Z_CD69:8_	49	ND	0.49
Z_CD69:10_	29	ND	0.78
Z_CD69:12_	30	ND	0.52

**Figure 4:**
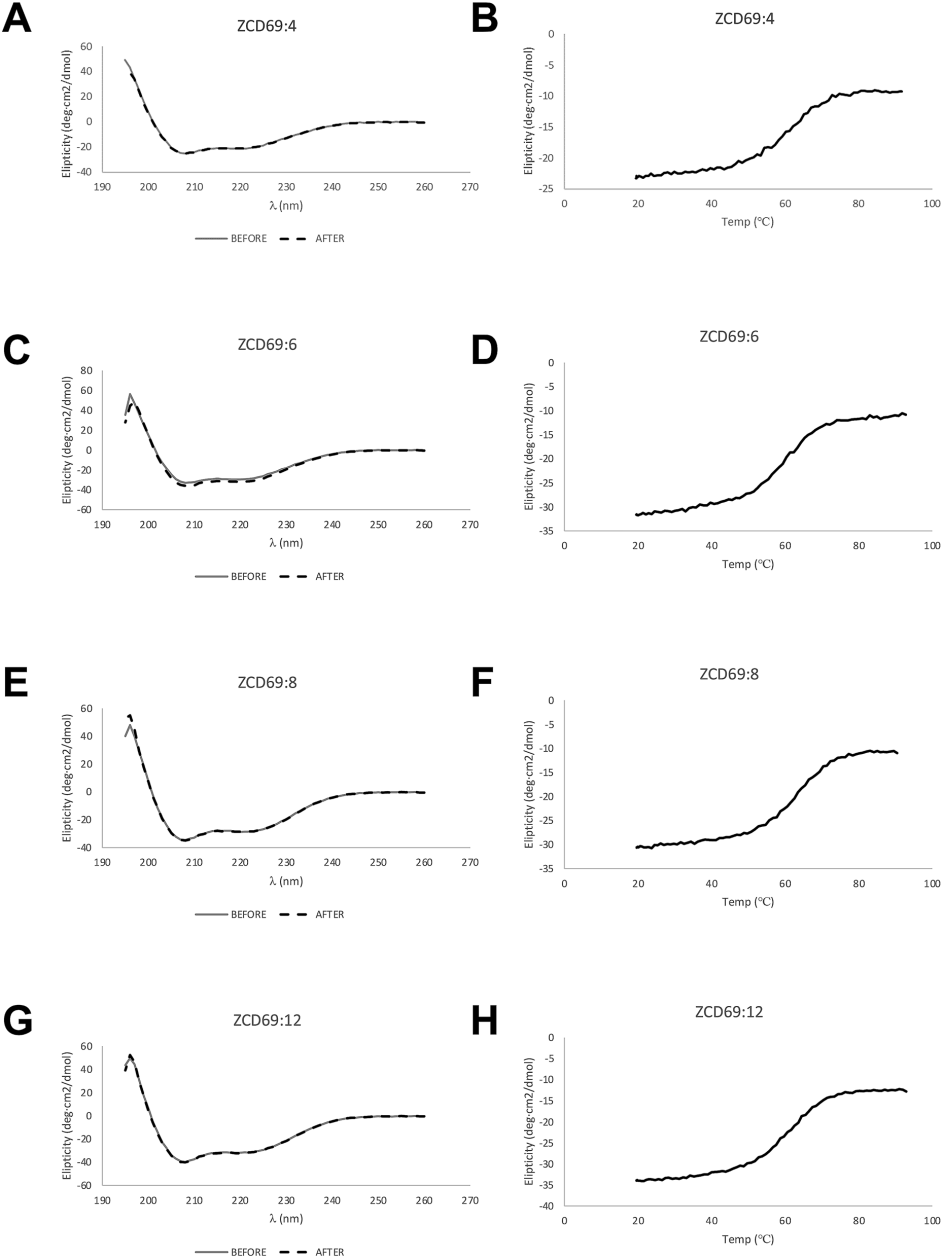
(**A**, **C**, **E**, **G**) Circular dichroism spectrum of affinity maturated Affibody molecules H_6_-Z_CD69:X_ at 20 °C before and after heat-induced denaturation, as well as (**B**, **D**, **F**, **H**) the result of thermal stability analysis of affinity maturated Affibody molecules H_6_-Z_CD69:X_ using circular dichroism spectroscopy.

The melting temperature for the five new Z variants was determined by circular dichroism spectroscopy and variable temperature measurements and were similar as for Z_CD69:2_ (Table 2). Measuring the CD spectrum before and after heat-induced denaturation demonstrated complete refolding for all variants (Fig. 4).

**Table 2:** Melting temperatures for tested Z variants from CD.

Tested Z variant in the format H6-Z_CD69:#_	Tm (°C)
Z_CD69:2_	62
Z_CD69:4_	59
Z_CD69:6_	59
Z_CD69:8_	62
Z_CD69:12_	60

### DOTA conjugation and radiolabeling and in vivo studies of the CD69-binding Z variants

The new variants, in the format of H_6_-Z_CD69:#-_Cys, were successfully conjugated with DOTA to a high degree as determined by MALDI (Supplementary Figs. 2–6), and subsequently purified using HPLC. All Z variants were radiolabeled, except for Z_CD69:10_ for which DOTA conjugation repeatedly exhibited low yields. Indium-111 chelation was successful for all other tested variants, i.e. for DOTA-Z_CD69:2_, DOTA-Z_CD69:4_, DOTA-Z_CD69:6_, DOTA-Z_CD69:8_ and DOTA-Z_CD69:12_, demonstrating acceptable yields and radiochemical purity (Table 3, Supplementary Figs. 7–11). The radiolabeled constructs were > 80% stable in formulation for up to 4 days.

**Table 3:** Results of radiolabeling tested DOTA-Z_CD69:#_ variants with indium-111.

Z variant as H_6_-Z_CD69:#-_DOTA	Added radioactivity (MBq)	Purity (%)	Molar Activity (MBq/nmol)	Radiochemical Yield (RCY) (%)	Stability
Z_CD69:2_	14	99	5	5	Not performed
Z_CD69:4_	85.0	99	6	71	91% (4 days)
Z_CD69:6_	92	98	9	90	98% (1 day)
Z_CD69:8_	76.5	100	6	84	88% (4 days)
Z_CD69:12_	83.2	99	18	93	67% (4 days)

^111^In-DOTA-Z_CD69:2_ retained binding to CD69 positive activated immune cells after radiolabeling (Supplementary Fig. 12).

**Figure 5:**
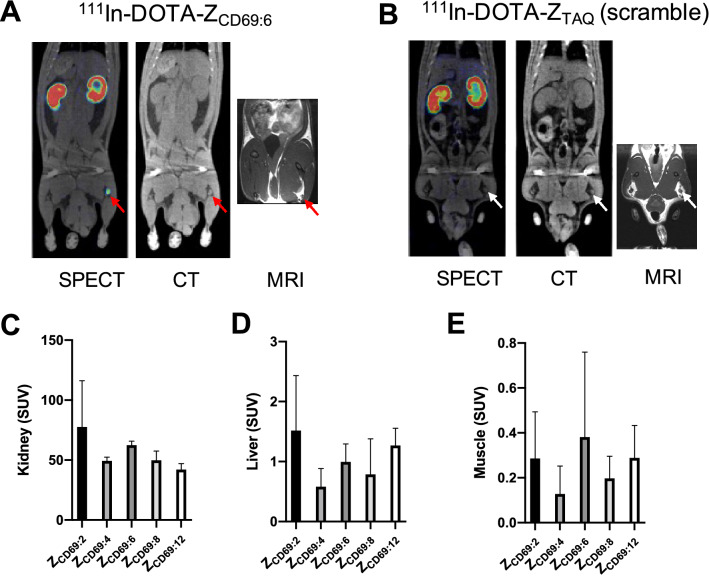
Representative coronal SPECT, CT and MRI images showing biodistribution and lymph node (red arrows) uptake 24 h post administration of ^111^In-DOTA- Z_CD69:6_ (**A**) and negative control ^111^In-DOTA- Z_TAQ_ (**B**). Bar diagrams showing uptake in kidney (48 h post administration, **C**), liver (3 h post administration, **D**) and muscle (3 h post administration, **E**) of the five different indium-111 labeled CD69 targeting variants, measured from SPECT/CT images (n = 3 rats each).

**Figure 6:**
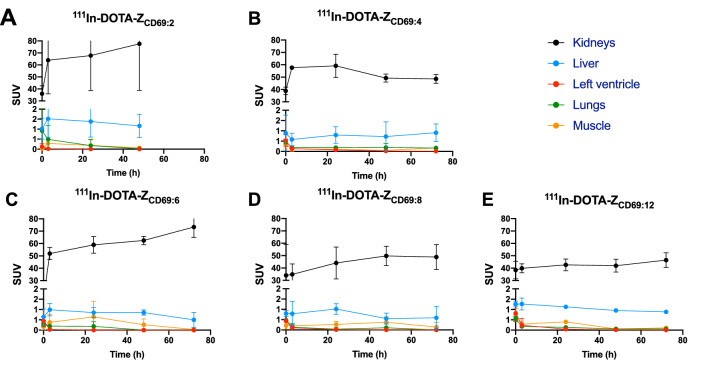
Dynamic uptake over time in kidney, liver, heart left ventricle (i.e. blood compartment), lungs and muscle tissue (average and standard deviations of n = 3 rats each) as assessed by SPECT/CT for ^111^In-DOTA-Z_CD69:2_ (**A**), ^111^In-DOTA-Z_CD69:4_ (**B**), ^111^In-DOTA-Z_CD69:6_ (**C**), ^111^In-DOTA-Z_CD69:8_ (**D**) and ^111^In-DOTA-Z_CD69:12_ (**E**).

After injection in rats, all indium-111 labeled Z variants exhibited rapid excretion though the kidneys and washout from most tissues (Fig. 5A–E, Fig. 6, Supplementary Figs. 13–17). The uptake and retention in the kidney cortex tended to differ between the Z variants, with ^111^In-DOTA-Z_CD69:2_ demonstrating the highest kidney uptake, followed by ^111^In-DOTA-Z_CD69:6_ (Fig. 5C). ^111^In-DOTA-Z_CD69:4_ and ^111^In-DOTA-Z_CD69:8_ had intermediate kidney uptake, while ^111^In-DOTA-Z_CD69:12_ exhibited the lowest uptake at the 48 h time point (Fig. 5C). For the background binding in liver and muscle tissue, ^111^In-DOTA-Z_CD69:4_ demonstrated the lowest binding, followed by ^111^In-DOTA-Z_CD69:8_ (Fig. 5D,E). ^111^In-DOTA-Z_CD69:2_, ^111^In-DOTA-Z_CD69:6_ and ^111^In-DOTA-Z_CD69:12_ all had higher background binding in liver and muscle (Fig. 5D,E). The control, non-binding affibody ^111^In-DOTA-Z_TAQ_ demonstrated a similar biodistribution pattern including renal excretion (Fig. 5B).

In two of the animals, the affinity matured variants also exhibited strong and sustained uptake in individual lymph nodes in the trunk and hind of the body (Fig. 5E). For example, in one animal examined with ^111^In-DOTA-_ZCD69:2_ exhibited sustained uptake in a left hind body lymph node (Supplementary Fig. 13), with SUV_mean_ ranging from 4 to 7 and SUV_max_ ranging from 23 to 55. One animal examined with ^111^In-DOTA-_ZCD69:6_ instead had sustained uptake in a right hind body lymph node (Supplementary Fig. 15), with SUV_mean_ ranging from 4 to 11 and SUV_max_ ranging from 20 to 57. The uptake in negative lymph nodes was generally SUV < 0.1. Lymph node binding was not seen for variants ^111^In-DOTA-Z_CD69:8_ or ^111^In-DOTA-Z_CD69:12_ (variants lacking affinity for murine CD69), nor for the control Affibody molecule ^111^In-DOTA-Z_TAQ_.

^111^In-DOTA-Z_CD69:2_ furthermore demonstrated binding at the site of allograft rejection in a mouse model (Supplementary data and Supplementary Fig. 18).

## Discussion

Inflammation is a biological response of the immune system that is involved either directly or indirectly in many human diseases. It is a defense mechanism that is crucial for survival, but when acute inflammation becomes uncontrollable, it may also damage the adjacent healthy cells. The improper resolution of inflammatory processes leads to a variety of chronic inflammatory diseases^15^. Due to the wide scale of inflammatory behavior in different types of diseases, more sensitive and specific biomarkers are required to improve prevention and treatment. For example, the understanding of the autoimmune response in several diseases is mainly based on animal models, human tissue biopsy or post-mortem analysis. Repeatable, quantitative and non-invasive methods for direct monitoring of the immune response in human would therefore potentially provide an important tool in diagnosis, management and treatment monitoring. In this study we report the discovery, optimization and radiolabeling of novel CD69 targeting Affibody molecules for quantitative assessment of inflammatory processes.

CD69 is expressed on most activated immune cells, especially in the early activation process. A general marker for activated immune cells is potentially more sensitive for detecting early subclinical immune responses than a cell-specific marker. Furthermore, the background binding of CD69 to resting circulating immune cells in blood is negligible in contrast to other putative immune cell imaging targets such as CD4 or CD8. Previous studies have reported that CD69 expression is often detected on cells in samples from inflamed tissues from patients with several different diseases, including T1D, rheumatoid arthritis, psoriasis, asthma, eosinophilic pneumonia, chronic obstructive pulmonary disease (COPD), chronic bronchitis, eosinophilic chronic rhinosinusitis (ECRS), arthritis, sarcoidosis, atopic dermatitis, atherosclerosis, systemic sclerosis, multiple sclerosis, systemic lupus erythematous, granulomatosis with polyangiitis (Wegener’s granulomatosis), neuromyelitis optica and autoimmune thyroiditis, as well in transplant biopsies of rejecting grafts and T and NK cells infiltrating tumors^4,6,7^. These clinical findings indicate that CD69 expression is a valid activation marker for all leukocytes in tissues with ongoing inflammation. Also, this indicates the potentially broad application of CD69 targeting imaging agents in many different diseases.

Here, Affibody molecules were developed and maturated with respect to affinity towards human CD69. All variants demonstrated acceptable stability and refolding properties as assessed by CD before and after heat denaturation. The refolding is especially important in the context of radiolabeling with e.g. radiometals, as the peptide often must be heated beyond the melting temperature for a short duration during radiolabeling. Misfolding after denaturation could generate a fraction of radiolabeled peptide with decreased binding capacity.

Furthermore, the Affibody molecules were evaluated with respect to binding affinity to human CD69, as well as murine CD69. Z_CD69:4_ in particular demonstrated improved affinity towards both human and murine CD69, compared to the primary variant Z_CD69:2_. Z_CD69:10_ and Z_CD69:12_ exhibited improved affinity to human CD69. However, Z_CD69:10_ and Z_CD69:12_ had poor affinity towards murine CD69, which would make preclinical evaluation of these constructs challenging. Thus, from these in vitro data, Z_CD69:4_ seemed as the most promising variant.

Affinity maturation of Affibody molecules can sometimes improve the affinity on the order of magnitudes. Here, affinity towards CD69 was only improved around twofold for a few of the variants. This outcome is less than optimal, but any increase in affinity will likely lead to a corresponding increase in sensitivity for an imaging probe. The reason for the limited improvement of affinity is unclear, but not without precedent for Affibody molecule maturation. A potential reason is the binding interface between the Affibody molecule (where two alpha helices with around 10–15 variable residues is responsible for binding) and the target protein. For the identified Z_CD69:#_ binders, only 8 residues vary, mainly in the first alpha helix (Supplemental Fig. 1). Perhaps there is not sufficient geometrical variability of the Affibody scaffold, to generate sub-nanomolar affinity binders specifically for the extracellular domain of CD69. In silico modelling is required to further understand the exact mode of action of binding of this new class of CD69 binders, but is outside of the scope of the current manuscript.

The new lead compound, Z_CD69:4_, exhibited an affinity towards both human and murine CD69 of approximately 30 nM, which is approaching the affinity of monoclonal affibodies, while having substantially small size and consequently faster in vivo targeting and clearance, critical for putative imaging probes.

To study the biodistribution, the variants were functionalized with a chelator, and radiolabeled with Indium-111. DOTA was, selected as it would potentially enable radiolabeling with both Indium-111 (SPECT) and Gallium-68 (Positron Emission Tomography, PET).

Z_CD69:10_ could not be reproducibly functionalized with DOTA for further radiolabeling, and thus discarded as a viable candidate. The radiochemical purity for all radiolabeled variants was high, in excess of 95%, and thus any signal contribution from other radiolabeled impurities can be considered negligible. As reference, a radiochemical purity in excess of 90%, with no single impurity > 5%, is often considered acceptable for clinical deliveries of PET radiopharmaceuticals.

The biodistribution of the radiolabeled Affibody molecules were evaluated by longitudinal SPECT-CT imaging, immediately from administration and for up to 72 h. The reason to use a longer half-life isotope ^111^In (2.8 days) in this study was to obtain longitudinal imaging biodistribution data by following the tracer kinetics for extended time period, as well as providing simpler logistics for the preclinical evaluation compared to e.g. Gallium-68.

The biodistribution and clearance of a radiolabeled construct is very important, as it determines when optimal imaging contrast can be obtained. The optimal scanning window in turn determines which radionuclides that can be used – as the radionuclide half-life must be in accordance with the optimal imaging time window. Here, we demonstrate rapid targeting and clearance of all radiolabeled Affibody molecules, i.e. low background is seen already at the 3 h time point. Importantly, the optimal imaging window is likely within 0–3 h after administration, which would enable future radiolabeling with some standard PET radionuclides including Gallium-68 (half-life 68 min) or Fluorine-18 (half-life 109 min). PET imaging has improved temporal and spatial resolution in comparison to SPECT, in addition to being quantitative, which is crucial when one attempts to measure activated immune cells over time.

As comparison, most radiolabeled larger peptides, such as antibodies, have slow clearance and an optimal imaging scanning time after a few days. The fast clearance observed with the Affibody molecules in this study highlights the importance of development of smaller peptide binders towards CD69, to enable quantitative PET imaging of this target. Furthermore, imaging with short lived (≈hours) PET radionuclides additionally yields a substantially lower radiation dose to the scanned patient, compared to radionuclides with a half-life of ≈days required for labeled antibodies. Radiation dose is especially critical when considering longitudinal imaging in young and relatively healthy populations, e.g. healthy control groups, individuals with T1D etc.

Radiolabeled peptides often exhibit renal clearance. Thus, renal uptake and retention should be minimized to reduce the predicted extrapolated absorbed radiation dose to the kidney. Therefore, we used low renal retention as one of the criteria for selecting the optimal Affibody molecule variant. Furthermore, the background binding of the radioligand should be minimized for optimal image contrast in lesions or tissues with activated CD69 expressing immune cells.

All tested Affibody molecule variants demonstrated acceptable kidney retention dose and background binding. However, also based on biodistribution, variant Z_CD69:4_ proved to be optimal candidate. Z_CD69:8_ and Z_CD69:12_ and exhibited similar or lower kidney dose than Z_CD69:4_, but on the other hand, their background binding in muscle and liver were higher. The differences between the groups in respect to kidney, liver and muscle binding were not statistically different based on a one-way ANOVA multiple-comparisons test, due to a few outliers (especially in the ^111^In-DOTA-Z_CD69:2_ group) as well as the relatively small group size (n = 3). However, the trend was robust in demonstrating improved biodistribution for e.g. ^111^In-DOTA-Z_CD69:4_ and ^111^In-DOTA-Z_CD69:8_ compared to the other variants. Further increase in group size was deemed as unnecessary for selecting the optimal variant for e.g. animal ethical considerations.

Although variant Z_CD69:4_ and Z_CD69:8_ demonstrated similarly suitable biodistribution, Z_CD69:8_ lacked affinity to murine CD69, which would potentially make preclinical evaluation challenging. Thus, of the variants with affinity to both murine and human CD69, Z_CD69:4_ exhibited the most beneficial renal and tissue background signal.

Some of the rats examined by SPECT/CT exhibited clearly detectable uptake of the CD69 binding variants in different lymph nodes across the body, especially ^111^In-DOTA-Z_CD69:2_ and ^111^In-DOTA-Z_CD69:6_. MRI scans was performed to further verify that the structures corresponded to lymph nodes (grey contrast tissue embedded in white adipose tissue, in MRI images in Fig. 5A,B). Importantly, the binding in individual lymph nodes was often sustained over several scans over up to 72 h (Supplementary Fig. 13 and 15), ruling out the possibility of an image artefact.

To explore if the lymph node binding was non-specific, we performed the same set of experiments but using a negative control affibody ^111^In-DOTA-Z_TAQ,_ with an amino acid sequence not binding to CD69. No detectable uptake was observed in any lymph nodes in either of the animals when using ^111^In-DOTA-Z_TAQ_, indication that affibodies don’t accumulate non-specifically in lymph nodes. Furthermore, several different affibody molecules have previously been radiolabeled and thoroughly examined in vivo in animals and humans, and focal uptake in lymph nodes are not generally observed. Thus, the lymph node binding of the CD69 binding variants is thus potentially an active and specific process. Additionally, lymph node binding was only seen for the variants with affinity for murine CD69 – lymph node targeting was not seen in rats administered with ^111^In-DOTA-Z_CD69:8_ or ^111^In-DOTA-Z_CD69:12_ (variants lacking affinity for murine CD69, Table 1). Potentially, the restricted binding in a lymph node in a certain part of the body is indicative of an immune response against local, subclinical infection. The apparent targeting of lymph nodes will be further explored in reproducible animal models of inflammation using the optimal variant.

In summary, variant Z_CD69:4_ displayed the optimal properties, both regarding stability, affinity and biodistribution, and was selected as lead compound for further development of a novel class of CD69 imaging agents. Future studies will focus on functionalization of Z_CD69:4_ to allow radiolabeling with positron emitting nuclides (e.g. ^18^F and ^68^ Ga) to generate a construct useful for in vivo PET imaging of CD69.

## Supplementary Information

Supplementary Information.
