# Supplementary material for: Discovery, optimization and biodistribution of an Affibody molecule for imaging of CD69
Source: Sci Rep. 2021 Sep 27;11:19151. doi: 10.1038/s41598-021-97694-6 (PMC8476556; doi:10.1038/s41598-021-97694-6)
Supplement: Supplementary file 1 — Supplementary Information. [file 41598_2021_97694_MOESM1_ESM.docx]

**Supplementary data**

**Discovery, optimization and biodistribution of an Affibody molecule for imaging of CD69**

**Materials and methods**

*Induction and verification of CD69 expression in human and murine immune cells*

Frozen PBMCs from healthy controls and mouse splenic cells were thawed and activated, for 24 hours in 37°C/CO_2_ 5%, with plate bound anti-CD3 (1µg/ml, anti-human clone OKT3, anti-mouse clone 145-2C11) in RPMI-1640 medium supplemented with L-glutamine 2mM, HEPES 10mM, penicillin-streptomycin 1% and 5% fetal calf serum (FCS). Control cells were cultured without anti CD3 stimulation at the same conditions. At 24 hours cells were harvested and put-on ice. Level of CD69 expression was assessed by FACS analysis. Briefly, small aliquots of stimulated and control cells were stained for CD45, CD3 and CD69 and acquired on a FACSVerse™ (BD Biosciences). Level of CD69 expression on leukocytes and T-cells were analyzed with FlowJo, BD Biosciences.

Antibodies used for phenotyping human cells were CD69-PECy7, clone FN50 (Biolegend), CD45-APC-H7, clone 2D1 (BD Biosciences) and CD3-Vio Blue, clone A6-13 (Miltenyi Biotec). Antibodies used for phenotyping mouse spleen cells were CD69-PECy7, clone H1.2F3 (Biolegend), CD45-AF488, clone 30-FII (Biolegend) and CD3-BV421, clone 17A2 (Biolegend).

*In vitro binding of ^111^In-DOTA-Z_CD69:2_ to activated immune cells*

^111^In-DOTA-Z_CD69:2_ was incubated with 0.3-2 million human peripheral blood mononuclear cells (PBMC) or mouse splenic cells, either non-activated or activated with plate bound anti-CD3 antibody (1µg/ml, clone OKT3). The fraction of CD69^+^ cells in each preparation was assessed by flow cytometry (see Supplementary materials for details).

Briefly, radioactive ^111^In-DOTA-Z_CD69:2_ (target amount 500 kBq corresponding to 10 nM peptide, incubation volume 1mL) was added to each cell suspension, and incubated for 1 h at 37°C. The cells were then washed and the supernatant was collected after centrifugation. The samples were measured in gamma counter (Wizard) as well as relevant controls (background, references). All samples were analyzed in triplicates. Afterwards the background activity and the remaining activity in the empty Eppendorf vials were measured separately. Cell binding was expressed as % of total incubated amount of ^111^In-DOTA-Z_CD69:2_ bound per million cells.

*In vivo imaging in mice with islet allograft*

Mice (n=5, NMRI, female, 30-34g) were transplanted with an islet allograft isolated from BALB/c mice. The islet isolation was performed according to standard procedures in our lab (Andersson, A. Isolated mouse pancreatic islets in culture: Effects of serum and different culture media on the insulin production of the islets. Diabetologia. 1978;14:397–404). The islets were administered to the recipient mice after administration analgesics and anaesthesia by isoflurane. The graft was injected subcutaneously on the left flank in a maximal volume of 100 µl. After implantation, the mice were allowed to wake up and returned to their cages. Five to six days later, when rejection of the graft was estimated as underway based on prior experience, the animals were examined by SPECT using ^111^In-DOTA-Z_CD69:2_ to visualize recruitment of activated immune cells at the site of allograft rejection.

Each animal was administered 0.6-0.7 MBq ^111^In-DOTA-Z_CD69:2_ in the tail-vein in a volume less than 100 µl. One mouse (day 5) was imaged by SPECT/CT repeatedly over the first 2 hours post injection (4 static whole-body scans, 30 minutes duration each), to determine the optimal imaging time-point post injection. The remaining mice (n=4) were imaged 6 days after transplantation with a single 30-minutes static whole-body examination 1-hour post-injection of ^111^In-DOTA-Z_CD69:2_.

**Results**

*In vitro binding of ^111^In-DOTA-Z_CD69:2_ to activated immune cells*

^111^In-DOTA-Z_CD69:2_ binding was higher in CD3 activated human PBMCs compared with resting PBMCs (Figure 1B) A similar increase in binding was also perceived in CD3 activated mouse splenic cells. ^111^In-DOTA-Z_CD69:2_ binding correlated well with the fraction of CD69^+^ cells in all cell preparations (R^2^=0.70, p<0.0001).

*In vivo imaging in mice with islet allograft*

^111^In-DOTA-Z_CD69:2_ displayed rapid renal excretion and low background in all mice, similarly to rats (Supplementary Figure 18). In the mouse examined at day 5 post-transplantation there was elevated uptake observed at the site of the allogenic islet graft (orange arrows, Supplementary Figure 18), but not at the contralateral subcutaneous site lacking a graft. The uptake around the islet graft was clearly visible in all 4 scans over 2 hours post-injection in this mouse, excluding that the accumulation was an image artefact. Previous experience with this allograft model indicates day 4-5 post-transplantation as the time when a strong local immune response should occur in the process of graft rejection. The mice which were examined with ^111^In-DOTA-Z_CD69:2_ on day 6 post transplantation displayed diffuse binding at the site of implantation, but of lower magnitude indicating lower amount of activated immune cells as the rejection process was subsiding or indicating the absence of active immune cells at the site following graft rejection.

**Supplementary Figures and Tables**

**Supplementary Figure 1.** The peptide sequences for the H_6_-Z_CD69:#-_Cys variants evaluated in the study.

**Supplementary Figure 2.** MALDI analysis of the DOTA conjugated Z_CD69:2_.

**Supplementary Figure 3.** MALDI analysis of the DOTA conjugated Z_CD69:4_.

**Supplementary Figure 4.** MALDI analysis of the DOTA conjugated Z_CD69:6_.

**Supplementary Figure 5.** MALDI analysis of the DOTA conjugated Z_CD69:8_.

**Supplementary Figure 6.** MALDI analysis of the DOTA conjugated Z_CD69:12_.

**Supplementary Figure 7.** Representative radiodetector (bottom) and UV (top) HPLC chromatograms following radiolabeling of Z variant Z_CD69:2_.

**Supplementary Figure 8.** Representative radiodetector (top) and UV (bottom) HPLC chromatograms following radiolabeling of Z variant Z_CD69:4_.

**Supplementary Figure 9.** Representative radiodetector (bottom) and UV (top) HPLC chromatograms following radiolabeling of Z variant Z_CD69:6_.

**Supplementary Figure 10.** Representative radiodetector (top) and UV (bottom) HPLC chromatograms following radiolabeling of Z variant Z_CD69:8_.

**Supplementary Figure 11.** Representative radiodetector (top) and UV (bottom) HPLC chromatograms following radiolabeling of Z variant Z_CD69:12_.

**Supplementary Figure 12.** Binding of ^111^In-DOTA-Z_CD69:2_ and correlation to expression of CD69 in anti-CD3 activated and resting human PBMCs and mouse splenic cells.

**Supplementary Figure 13.** Biodistribution of ^111^In-DOTA-Z_CD69:2_ at several timepoints after administration, in a representative healthy rat. The figure shows SUV corrected coronal projections at the level of the hind body lymph-nodes and when possible the kidneys.

**Supplementary Figure 14.** Biodistribution of ^111^In-DOTA-Z_CD69:4_ at several timepoints after administration, in a representative healthy rat. The figure shows SUV corrected coronal projections at the level of the hind body lymph-nodes and when possible the kidneys.

**Supplementary Figure 15.** Biodistribution of ^111^In-DOTA-ZCD69:6 at several timepoints after administration, in a representative healthy rat. The figure shows SUV corrected coronal projections at the level of the hind body lymph-nodes and when possible the kidneys.

**Supplementary Figure 16.** Biodistribution of ^111^In-DOTA-ZCD69:8 at several timepoints after administration, in a representative healthy rat. The figure shows SUV corrected coronal projections at the level of the hind body lymph-nodes and when possible the kidneys.

**Supplementary Figure 17.** Biodistribution of ^111^In-DOTA-ZCD69:12 at several timepoints after administration, in a representative healthy rat. The figure shows SUV corrected coronal projections at the level of the hind body lymph-nodes and when possible the kidneys.

**Supplementary Figure 18.** Accumulation of ^111^In-DOTA-Z_CD69:2_ at the site of an islet allograft (yellow arrow) in mouse. The inferior part of the kidneys (red arrows) and the bladder (grey arrow) are also indicated. Both coronal (left) and transaxial (right) projections are shown.

**Supplementary Table 1.** Design of Z variant library for affinity maturation selection

| Position in the Z variant sequence | Permitted amino acid residues | Proportions |
| --- | --- | --- |
| 9 | A,D,E,F,G,I,K,L,M,N,Q,R,S,T,V,W,Y | F: 70%; Rest: 1.9% each |
| 10 | A,D,E,F,G,I,K,L,M,N,Q,R,S,T,V,W,Y | Y: 70%; Rest: 1.9% each |
| 11 | A,D,E,F,G,I,K,L,M,N,Q,R,S,T,V,W,Y | H: 70%; Rest: 1.9% each |
| 13 | A,D,E,F,G,I,K,L,M,N,Q,R,S,T,V,W,Y | M: 70%; Rest: 1.9% each |
| 14 | A,D,E,F,G,I,K,L,M,N,Q,R,S,T,V,W,Y | K: 70%; Rest: 1.9% each |
| 17 | A,D,E,F,G,I,K,L,M,N,Q,R,S,T,V,W,Y | L: 70%; Rest: 1.9% each |
| 18 | A,D,E,F,G,I,K,L,M,N,Q,R,S,T,V,W,Y | K: 70%; Rest: 1.9% each |
| 24 | A,D,E,F,G,I,K,L,M,N,Q,R,S,T,V,W,Y | K: 70%; Rest: 1.9% each |
| 25 | A,D,E,F,G,I,K,L,M,N,Q,R,S,T,V,W,Y | Y: 70%; Rest: 1.9% each |
| 27 | A,D,E,F,G,I,K,L,M,N,Q,R,S,T,V,W,Y | K: 70%; Rest: 1.9% each |
| 28 | A,D,E,F,G,I,K,L,M,N,Q,R,S,T,V,W,Y | E: 70%; Rest: 1.9% each |
| 31 | D,H,I,K,Y | K:60%; D:10%; H:10%; I:10%; Y:10% |
| 32 | A,D,E,F,G,I,K,L,M,N,Q,R,S,T,V,W,Y | T: 70%; Rest: 1.9% each |
| 35 | A,D,E,F,G,I,K,L,M,N,Q,R,S,T,V,W,Y | K: 70%; Rest: 1.9% each |

**Supplementary Table 2.** Conditions for Indium-111 radiolabeling of the H6-Z_CD69:#-_DOTA variants.

| Z variant as H6-Z_CD69:#-_DOTA | μl 111In solution | MBq 111In | nmol Z variant added | Reaction time | Buffer | pH | Purification |
| --- | --- | --- | --- | --- | --- | --- | --- |
| Z_CD69:2_ | 280 | 14 | 3 | 30 min | sodium acetate | 5.5 | SPE |
| Z_CD69:4_ | 150 | 156 | 13.9 | 1 h | ammonium acetate | 5.2 | NAP-5 |
| Z_CD69:6_ | 300 | 103 | 10 | 30 min | HEPES (0.1 M) | 5.0 | SPE |
| Z_CD69:8_ | 120 | 120 | 13.4 | 1 h | ammonium acetate | 5.5 | NAP-5 |
| Z_CD69:12_ | 100 | 102 | 4.7 | 30 min | ammonium acetate | 5.5 | NAP-5 |
